# Investigation of the Local Recurrence Rate after Colorectal Endoscopic Mucosal Resection: Is Incomplete Polyp Resection Really a Clinically Important Problem? Analysis of the Rationale for the “Resect and Discard” Strategy

**DOI:** 10.1155/2019/7243515

**Published:** 2019-01-08

**Authors:** Jun Arimoto, Takuma Higurashi, Hideyuki Chiba, Noboru Misawa, Tsutomu Yoshihara, Takayuki Kato, Kenji Kanoshima, Akiko Fuyuki, Hidenori Ohkubo, Takashi Nonaka, Takamitsu Sato, Eiji Sakai, Hiroshi Iida, Tohru Goto, Atsushi Nakajima

**Affiliations:** ^1^Department of Gastroenterology, Omori Red Cross Hospital, Tokyo, Japan; ^2^Department of Gastroenterology and Hepatology, Yokohama City University School of Medicine, Yokohama, Japan; ^3^Department of Gastroenterology, National Yokohama Medical Center, Yokohama, Japan; ^4^Department of Gastroenterology, Kanto Medical Center NTT EC, Tokyo, Japan

## Abstract

**Background/Aims:**

The “Resect and Discard” strategy is a potentially useful strategy. At present, only the lesion size and accuracy of diagnosis are cited as considerations for clinical adoption of this strategy. On the other hand, histopathology of the resected specimens after Endoscopic Mucosal Resection (EMR) reveals often an unclear or positive-margin status, implying Incomplete Polyp Resection (IPR). If IPR indeed increased the risk of local recurrence, histopathological evaluation of the margin would be indispensable and clinical adoption of this strategy is difficult. The aim of this study is to verify the association between IPR and the risk of local recurrence.

**Methods:**

The 1872 polyps and 603 EMR cases in 597 patients who had EMR between May 2013 and May 2014 were enrolled. The local recurrence rate until 3 years after the EMR in cases with the target lesions of the “Resect and Discard” strategy was determined in the negative-margin and IPR groups.

**Results:**

The final analysis was performed using the data of 1092 polyps, and 222 were categorized into the IPR group. There were no cases of recurrence in either of the groups.

**Conclusion:**

This is the world's first report conducted to examine the correlation of IPR and the local recurrence rate for clinical practice of “Resect and Discard” strategy. There is the possibility that pathological evaluation of the margins after EMR in patients with small polyps can be skipped.

## 1. Introduction

The incidence of colorectal cancer (CRC) continues to increase around the world [1, 2], and the importance of early detection and early treatment is growing. Endoscopic Mucosal Resection (EMR) of colorectal polyps has been shown to be associated with a significant reduction in the risk of CRC [3–8]. As EMR of colorectal polyps is an effective treatment for prevention of CRC, early detection and early resection of colorectal polyps is one of the fundamental strategies for reducing CRC mortality. However, an important issue associated with early detection and early resection of colorectal polyps is escalation of the medical costs and increase in time and labor required for formal histopathological diagnosis of the large number of resected polyps resulting from the increased detection rates.

In recent years, some studies have reported the potential usefulness and feasibility of the “Resect and Discard” strategy in clinical practice [9–11]. The potential cost saving of not sending diminutive polyps for formal histopathology is thought to exceed $95 million per year in the United States alone [12, 13]. The benefit of this strategy is not limited to cost reduction but also includes savings in labor and pathology time [14]. Although the target lesions for this strategy should be selected carefully [15], this strategy has numerous potential benefits associated with the practice of early detection and resection of colorectal polyps for reducing CRC mortality.

At present, only small polyps (less than 10 mm in diameter) and ≥90% accuracy of optical diagnosis with high confidence are cited as indications for adoption of the “Resect and Discard” strategy [9, 10, 16]. However, we believe that, in addition to these two criteria, the influence of the pathological margin status on the risk of local recurrence after EMR is also an important consideration in establishing the rationale for clinical adoption of this strategy. It would be faulty to recommend this strategy without first undertaking a discussion on the association between the pathological margin status and the risk of local recurrence after EMR. Histopathology of the resected specimens after EMR often reveals a positive or unclear tumor margin status, implying Incomplete Polyp Resection (IPR). There are some reports to suggest that IPR is a risk factor for Interval colorectal cancer (ICC) development [17–19]. If IPR were indeed a risk factor for ICC, evaluation of the pathological margins after EMR would be indispensable, and it would be difficult to recommend clinical adoption of the “Resect and Discard” strategy. The aim of this study was to evaluate the association of the pathological margin status with the risk of local recurrence after EMR, to establish the rationale for clinical adoption of this strategy.

## 2. Methods

### 2.1. Study Design and Data Collection

**Figure 1:**
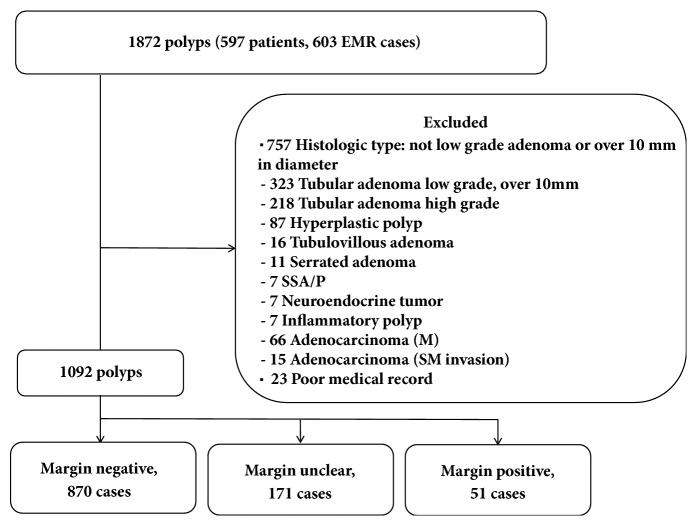
Flow diagram of this study. After EMR, each polyp was categorized into the negative-margin group, unclear-margin group, or positive-margin group.

This retrospective cohort study was performed at the Department of Gastroenterology and Hepatology, Yokohama City University Hospital, Yokohama, Japan. We identified consecutive patients with colorectal polyps who underwent EMR between May 2013 and May 2014 from our prospectively maintained database and enrolled them for this analysis. The coordinating office was at Yokohama City University Hospital, with the registration and data collection conducted at this site. Subject enrollment began in February 2017, and the study was completed in June 2017. The patient inclusion criteria were as follows: (a) at least one surveillance colonoscopy had been performed within 3 years after EMR; (b) complete colonoscopy (colonoscopy reaching the cecum) had been performed at every examination; (c) the final diagnosis was low-grade tubular adenoma; (d) the lesion was less than 10 mm in diameter. Patients diagnosed by histopathology as having hyperplastic polyp, high grade tubular adenoma, tubulovillous adenoma, serrated adenoma, SSA/P, neuroendocrine tumor, inflammatory polyp, adenocarcinoma were excluded; we only included patients with target lesions for the “Resect and Discard” strategy. In this study, all polyps were removed by EMR. The polyps were categorized according to the pathological margin status after EMR into the negative-margin group, unclear-margin group, or positive-margin group (Figure 1). We investigated the local recurrence rates in each of these three groups.

### 2.2. Data Analysis and Definition of IPR

The primary endpoint was the correlation between the local recurrence rate and the pathological margin status after EMR. Cases where both the lateral and deep margins were free of tumor cells were classified into the negative-margin group. Cases where it was unclear whether resection margins were involved or not were classified into the unclear-margin group. Cases where any of the lateral or deep margins contained tumor cells were classified into the positive-margin group. Finally, cases of the unclear-margin group and positive-margin group were sorted into the IPR group. We investigated the local recurrence rate at 1, 2 and 3 years after EMR for each margin status in each of the groups. The size of the polyp was estimated by the endoscopist during the EMR and measured precisely by the pathologist after EMR in the resected specimen. The tumor location was estimated using anatomic landmarks (such as ileocecal (IC) valve, hepatic flexure (HF), sigmoid-descending (SD) junction, and rectosigmoid (RS) junction) and the insertion distance upon withdrawal of the endoscopy.

### 2.3. Ethical Considerations and Registration

The study protocol was in compliance with the Declaration of Helsinki and the Ethics Guidelines for Clinical Research published by the Ministry of Health, Labour and Welfare of Japan. Approval for this study was obtained from the Ethics Committee of Yokohama City University Hospital on 22nd February, 2017.

### 2.4. Endoscopic Procedure

The bowel preparation was initiated from the day prior to the EMR. Each patient was instructed to consume a low-residue diet and take 5 mg of oral sodium picosulfate on the evening before the procedure. On the day of the EMR, the patients received 2000 ml of polyethylene glycol (PEG). If the stools were not sufficiently clear, an additional 1000-2000 ml of PEG was given to ensure sufficient bowel cleaning. We used either conventional or magnifying endoscopes (CF-Q260AI, CF-H260AZI, PCF-Q260AZI, CF-HQ290I; Olympus, Tokyo, Japan). If necessary, midazolam was used for sedating the patient, and the cardiorespiratory function was monitored during the procedure. All procedures were performed with a CO_2_ insufflation system. The VIO300D (ERBE Elektromedizin, Tuebingen, Germany) power source was used for the electrical cutting and coagulation, and the polyp was removed with snares (Olympus) after saline injection mixed with indigo carmine. After the EMR, we carefully observed the wound in all cases using the white light and narrow-band imaging (NBI) endoscopy modes to confirm that there was no residual lesion.

### 2.5. Statistical Analysis

The results are presented as means or medians (±standard deviation or range) for the quantitative data, and as frequencies (percentage) for the categorical data. Categorical data were analyzed using the *χ*^2^ test or Fisher's exact test, as appropriate. Data showing normal distribution were compared by the t-test and those showing nonnormal distribution were compared by the Mann-Whitney *U* test, to assess the statistical significance of differences. P <0.05 was considered as denoting statistical significance. All statistical analyses were carried out using SPSS statistics, version 18 (SPSS, Chicago, IL, USA).

## 3. Results

### 3.1. Patient and Polyp Characteristics

We performed EMR in 597 patients (603 EMR cases, 1872 polyps) between May 2013 and May 2014. We excluded 780 polyps from the analysis, with the final analysis was performed on 1092 polyps (Figure 1). Depending on the histological status of the resection margin, the polyps were divided into three groups: the negative-margin group (870 polyps), unclear-margin group (171 polyps) and positive-margin group (51 polyps). The 222 polyps (including 171 of the unclear-margin group and 51 of the positive-margin group) were classified into the IPR group. The patient and polyp characteristics are presented in Table 1. The patients consisted of 388 men and 135 women with a mean age (±SD) of 67 (±9.9) years. In the majority of cases, the polyps were located in the ascending colon, transverse colon or sigmoid colon. In regard to the polyp size, 53.4% of the polyps (584/1092) were ≤5 mm in diameter, and the tumor morphology was classified as Isp or Is in the majority of cases. There were no significant differences in the polyp characteristics (location, size and morphology) between the margin-negative group and the IPR group.

### 3.2. Local Recurrence after EMR

The local recurrence rates at 1, 2, and 3 years after EMR for each margin status are presented in Table 2. There were no cases with recurrence until 3 years after EMR, in either the negative-margin or the IPR group. Namely, even in the positive-margin group, there were no cases of local recurrence until 3 years after EMR. The mean incidence density of the local recurrence in the margin-positive group was 0 case/270 polyp-years) and the mean incidence density of the local recurrence in the IPR group was 0 case/740 polyp-years).

## 4. Discussion

This is the world's first report of a study conducted to examine the association between IPR and the risk of local recurrence towards establishing the rationale for adoption of the “Resect and Discard” strategy in clinical practice. The American Society of Gastrointestinal Endoscopy (ASGE) published the “Preservation and Incorporation of Valuable Endoscopic Innovation (PIVI)” statement on real-time endoscopic assessment of the histology of diminutive colon polyps [15]. According to the PIVI statement, polyps measuring ≤5 mm in diameter could represent target lesions for this strategy. On the other hand, Takeuchi et al. [14] reported that polyps less than 10 mm in diameter could be target lesions for the “Resect and Discard” strategy by using magnifying NBI for endoscopic diagnosis. In addition to these criteria, the influence of the pathological margin status on the risk of local recurrence is also an important consideration in establishing the rationale for clinical adoption of this strategy. We examined whether IPR in cases with small low-grade adenomas was actually associated with an increase in the local recurrence rate in the region corresponding to the site of the EMR and whether IPR in cases with such lesions was truly of any clinical importance.

There are some reports to suggest that IPR is a risk factor for ICC development [17–19]. If IPR were indeed a risk factor for ICC, evaluation of the pathological margins after EMR would be indispensable, and it would be difficult to recommend clinical adoption of the “Resect and Discard” strategy. However, it still remains unclear whether IPR is actually an independent risk factor for ICC for the target lesions of this strategy, namely, small polyps measuring less than 10 mm in diameter. Pohl H et al. reported that the risk of IPR is affected by the polyp size, polyp histology (e.g., sessile serrated histology), and the skill level of the endoscopist [20]. Lee SP et al. reported that location of the polyp in the proximal part of the colon and rectum, advanced polyps, and inadequate experience of the assistant in performing EMR were risk factors for IPR [21]. However, these factors can also be risk factors for ICC development. Therefore, there is the possibility that not IPR* per se,* but the above-mentioned factors (large polyp size, sessile serrated histology, and advanced polyps) are the actual risk factors for ICC development. In addition, missed adenoma/carcinoma and de novo cancer formation from flat and depressed lesions are also considered as risk factors for ICC development [22–26], and it still remains under debate whether IPR per se represents an independent risk factor for ICC development. Furthermore, there is the possibility that in cases with small polyps and nonadvanced polyps, IPR may not be a risk factor for ICC development. As the target lesions for the “Resect and Discard” strategy consist of small polyps measuring less than 10 mm in diameter, it is possible that IPR of these lesions may have no adverse bearing on clinical adoption of the “Resect and Discard” strategy. Therefore, in this study, we targeted small polyps measuring less than 10 mm in diameter to verify the validity of adoption of this strategy for these lesions.

Our results revealed no cases of local recurrence until 3 years after EMR, in either the negative-margin group or the IPR group. Namely, even in the positive-margin group, there were no cases of local recurrence until 3 years after EMR. The following possibility is speculated for explaining these results. The possibility is the burn effect in positive-margin cases. There is the possibility that tumor cells remaining in the colon are necrotized by the burn effect, which could have prevented local recurrence in some cases of the IPR group. Our findings in this study provide a rationale for clinical adoption of the “Resect and Discard” strategy. We demonstrated that IPR is not a risk factor for local recurrence in cases with small polyps measuring less than 10 mm in diameter. In other words, for such lesions, there is the possibility that pathological evaluation of the margins after EMR can be skipped. The two conditions of high accuracy of endoscopic diagnosis plus the lack of an association between the pathological margin status and the risk of local recurrence provide the rationale, for the first time, for clinical adoption of the “Resect and Discard” strategy. This study provides indispensable data encouraging adoption of this strategy in the future, and the results represent an ethical guarantee for adoption of this strategy. We could say that this study has helped us take a big step towards clinical adoption of the “Resect and Discard” strategy.

Our study had some limitations. First, the data were collected retrospectively from medical records. The resection of small polyp does not leave a visible scar in many cases, and the site of resection is difficult to relocate. Therefore, we cannot exclude the possibility of have missed some cases of local recurrence. It would be more desirable to recruit patients prospectively and carry out similar evaluation of the local recurrence rates in cases with negative and IPR margin status. Second, this study was based on a single-center experience, and there is the possibility of bias in the evaluation of the pathological margins by the pathologists at the center. If the pathologists were to select one of only two options (negative or positive), the results could have been affected by judgment bias of the pathologists. However, as there was the third option of “unclear margin” for lesions that were difficult to judge as positive or negative, we believe that there was no significant effect of judgment bias on the results. As there were no cases of local recurrence in any of the groups anyway, any bias in the evaluation of the pathological margins would be rendered irrelevant. Thus, regardless of the pathological margin status, there were no cases of local recurrence among polyps for which endoscopic curative resection had been confirmed. Third, all polyps were removed by EMR. Polyps removed by Cold Snare Polypectomy (CSP) were not included; the results of this study cannot be applied to polyps removed by CSP. CSP techniques are widely spread for the resection of polyps [27], and the European Society of Gastrointestinal Endoscopy guideline recommend CSP as the first choice for small polyps [28]. However, we think that polyps removed by EMR and polyps removed by CSP should not be considered together in one study. In the cases of EMR, it might be assumed that burn effect can prevent recurrence. The presence or absence of burn effect is the difference between EMR and CSP, we think that it is inaccurate to examine the association of the pathological margin status with the risk of local recurrence after EMR and that of CSP in one study. In regard to the pathological margin status after CSP, we would like to evaluate in the next study. Fourth, only followed up our cases for 3 years, and there is the possibility of local recurrence developing at a later time, e.g., after 4 years or more. However, we demonstrated absence of local recurrences for at least 3 years after EMR, and in such cases, significant local recurrence is unlikely to develop after 4 years or more. Therefore, we believe that our results and conclusion are unlikely to have been biased by the relatively short follow-up period of 3 years. Fifth, we excluded high-risk lesions (i.e., high grade adenoma, tubulovillous adenoma). It is possible to happen that it was actually a high-risk lesion that was diagnosed as a low-grade adenoma at the time of endoscopic diagnosis (the target lesion of the “Resect and Discard” strategy). We think that further examination is necessary regarding this point. Taking into consideration these limitations, we would like to verify our results by conducting a prospective multicenter study in the future.

In conclusion, there were no cases of local recurrence until 3 years after EMR in either the negative-margin or the IPR group. Our results indicated the absence of a correlation between the risk of local recurrence and the pathological margin status after EMR. There is the possibility that pathological evaluation of the margins after EMR in patients with small polyps can be skipped. We consider that this study offers useful evidence to encourage clinical adoption of the “Resect and Discard” strategy in the future.

## Figures and Tables

**Table 1:** Patient and polyp characteristics.

Patients characteristics					

Sex					
Male					388 (74%)
Female					135 (26%)
Age (years) mean ± SD (range)					67 ± 9.9 (21-87)

Polyp characteristics	Negative	Unclear	Positive	IPR	p-value

Location					N.S
IC valve (4)	4	0	0	0	
Cecum (61)	36	18	7	25	
Ascending colon (233)	182	39	12	51	
Hepatic flexure (35)	23	12	0	12	
Transverse colon (237)	203	29	5	34	
Splenic flexure (2)	1	0	1	1	
Descending colon (140)	118	17	5	22	
SD junction (3)	3	0	0	0	
Sigmoid colon (305)	252	39	14	53	
Rs junction (28)	19	7	2	9	
Ra (20)	12	4	4	8	
Rb (24)	17	6	1	7	
Size					N.S
≤5 mm (584)	466	85	33	118	
6-9 mm (508)	404	86	18	104	
Morphology					N.S
Ip (94)	83	9	2	11	
Isp (507)	410	76	21	97	
Is (453)	351	79	23	102	
IIa (38)	26	7	5	12	

N.S: not significant.

**Table 2:** The incidence density of local recurrence for each margin status.

Follow-up period	Negative	Unclear	Positive	IPR
1-year (1092)	0% (0/870)	0% (0/171)	0% (0/51)	0% (0/222)
2-years (470)	0% (0/352)	0% (0/82)	0% (0/36)	0% (0/118)
3-years (396)	0% (0/302)	0% (0/66)	0% (0/28)	0% (0/94)
Total (1092)	0% (0/870)	0% (0/171)	0% (0/51)	0% (0/222)
Cases per Polyp-years	0/2480	0/533	0/207	0/740

## Data Availability

All data used to support the findings of this study are available from the corresponding author upon request.

## Abbreviations

CRC: Colorectal cancer
EMR: Endoscopic mucosal resection
IPR: Incomplete polyp resection
ICC: Interval colorectal cancer
ESD: Endoscopic submucosal dissection
IC: Ileocecal
HF: Hepatic flexure
SD: Sigmoid-descending
RS: Rectosigmoid
STROBE: Strengthening the Reporting of Observational Studies in Epidemiology
PEG: Polyethylene glycol
NBI: Narrow-band imaging
ASGE: American Society of Gastrointestinal Endoscopy
PIVI: Preservation and Incorporation of Valuable Endoscopic Innovation
CSP: Cold Snare Polypectomy.

## Supplementary Materials

Supplementary Materials STROBE Statement—Checklist of items that should be included in reports of cohort studies.
